# A hip extensor–assistive soft exosuit training redistributes plantar pressure in older adults: a randomized controlled pilot trial

**DOI:** 10.3389/fbioe.2026.1857758

**Published:** 2026-07-16

**Authors:** Na Young Yun, Jaeha Yang, Kyeongmin Lim, Sejun Park, Giho Lee, Byeongjun Cho, Giuk Lee, Ho Seok Lee, Don-Kyu Kim, Hyun Iee Shin

**Affiliations:** 1 Department of Physical Medicine and Rehabilitation, College of Medicine, Chung-Ang University, Seoul, Republic of Korea; 2 Department of Physical Medicine and Rehabilitation, Chung-Ang University Hospital, Seoul, Republic of Korea; 3 School of Mechanical Engineering, Chung-Ang University, Seoul, Republic of Korea; 4 HUROTICS Inc., Seoul, Republic of Korea; 5 Department of Physical Medicine and Rehabilitation, Chung-Ang University Gwangmyeong Hospital, Gwangmyeong-si, Republic of Korea

**Keywords:** gait, hip extensor assistance, older adults, plantar pressure, rehabilitation, robot assisted gait training, soft wearable robotic suit

## Abstract

**Background:**

Age-related gait decline is associated with reduced heel loading, forefoot overloading, and impaired gait stability. Soft wearable robotic suits that assist hip extension may improve gait biomechanics, but evidence for training-induced carry-over effects on plantar pressure distribution remains limited in community-dwelling older adults. This study investigated whether a short-term gait training program using a hip extensor-assistive soft wearable robotic suit could modify plantar pressure and spatiotemporal gait parameters under device-free assessment conditions.

**Methods:**

In this prospective, single-center, randomized controlled pilot trial, 42 community-dwelling older adults aged 65–79 years were randomly assigned to a robot-assisted training group (n = 21) or a control group (n = 21). Both groups completed a 3-week intervention consisting of six sessions, each including 20 min of treadmill-based gait training and 20 min of strength training. The robot-assisted group wore a cable-driven soft wearable robotic suit providing bilateral hip extension assistance during treadmill training, whereas the control group performed identical training without the device. All post-intervention assessments were conducted without the device. Plantar pressure and spatiotemporal gait parameters were measured using the GAITRite® system at baseline and post-intervention.

**Results:**

Compared with the control group, the robot-assisted group showed a greater increase in heel contact area (p = 0.014), greater reductions in forefoot contact area (p = 0.016) and forefoot peak plantar pressure (p = 0.026), and significant time × group interactions for heel contact area (F = 7.273, p = 0.011), forefoot contact area (F = 5.042, p = 0.031), and forefoot peak plantar pressure (F = 5.535, p = 0.024). The robot-assisted group also showed reduced step length (p = 0.005), stride length (p = 0.005), single support time (p = 0.032), and swing phase duration (p = 0.037), along with increased stance phase duration (p = 0.041).

**Conclusion:**

Short-term training with a hip extensor-assistive soft wearable robotic suit was associated with changes in plantar loading and gait cycle patterns under device-free assessment conditions, suggesting that gait pattern modifications may occur after repeated training sessions.

## 1 Introduction

With the global aging population, extending the healthy life expectancy of older adults is an important societal challenge (Gianfredi et al., 2025). Gait is a fundamental indicator of successful aging, and its impairment—affecting over 60% of individuals aged 80 and older—directly leads to reduced mobility, falls, and a decline in quality of life (Hoff et al., 2025; Pirker and Katzenschlager, 2016).

A primary biomechanical cause for this age-related gait decline is the weakening of ankle plantarflexors (Pol et al., 2021). To compensate for reduced distal power, older adults instinctively adopt a distal-to-proximal shift strategy, relying on their hip extensors for forward propulsion (Browne and Franz, 2019; DeVita and Hortobagyi, 2000). However, when the hip extensors also lack sufficient eccentric control and power, this compensatory mechanism is limited. Consequently, older adults adopt a shuffling, stability-oriented gait strategy driven by a fear of falling, characterized by inadequate leg extension and stepping with the entire foot (Susilo et al., 2025).

These proximal deficits fundamentally disrupt the distal foot strike pattern, leading to an inefficient dynamic plantar pressure distribution (Browne and Franz, 2019). The disruption of normal heel loading patterns has been associated with excessive concentration of plantar pressure on the forefoot. This pathologically prolonged forefoot loading time and high peak pressure on the forefoot not only impair gait efficiency but exacerbate instability during weight acceptance, increasing the risk of falls (Pol et al., 2021; Yan et al., 2023). Therefore, restoring proximal joint (hip) function is important to distal (plantar pressure) mechanics.

To address these issues, soft wearable robotic suits offer a customized intervention (Martinez-Hernandez et al., 2021; Zhu et al., 2022). Unlike conventional exoskeleton or end-effector robots, which can be bulky and limits natural gait trajectories or misalignments with joint rotation centers, the core design of soft exosuits focuses on a flexible, clothing-like structure without rigid joints (Tricomi et al., 2024). This provides improved comfort and preserves kinematic autonomy (Kim et al., 2024; Han et al., 2025). In particular, cable-driven soft wearable robots incorporating Bowden cables transmit force through a motor pulling the cable, thereby providing precise assistance to the target joints (Fang et al., 2021).

Providing localized assistance to a single targeted joint can induce an indirect assistance effect (Franks et al., 2021). By assisting the hip, the user adapts their overall gait pattern, thereby optimizing the muscular coordination and biomechanical efficiency of the unassisted distal joints, such as the ankle (Franks et al., 2021). Enhancing hip extension increases forward propulsion and facilitates an efficient stance-to-swing transition, which is necessary for clearing the foot safely (Wonsetler et al., 2018).

Previous gait rehabilitation studies have often focused on increasing gait speed and step length as the main indicators of improvement (Lee et al., 2023; Cho et al., 2025)**.** However, an increase in gait speed alone does not necessarily mean functional recovery (Cesari, 2011). When speed increases without sufficient dynamic stability, the risk of falls may actually increase. Evidence suggests that spatiotemporal gait variability, rather than absolute gait speed alone, is more closely associated with fall risk in older adults (Maki, 1997).

Older adults naturally tend to adopt a more stability-oriented gait strategy. Therefore, rehabilitation should consider not only quantitative changes but also qualitative reorganization of the gait cycle (Susilo et al., 2025). Despite this need, it remains unclear how external proximal assistance influences distal mechanics during gait, particularly plantar pressure distribution, to enhance gait stability. Furthermore, long-term robot-assisted gait training programs may place an economic burden on community-dwelling older adults and lead to reduced compliance with high-frequency sessions. Therefore, examining whether a short-term, minimum-frequency protocol can still produce meaningful improvements in gait biomechanics would provide an important clinical basis.

We hypothesized that a three-week training program with a cable-driven, hip extension–assistive soft robotic suit would alter the gait of older adults. We expected plantar loading to become more heel-centered. These changes were thought to reflect enhanced proximal stability (Pirker and Katzenschlager, 2016; Wonsetler et al., 2018). This study examined plantar pressure distribution and gait cycle phasing as biomechanical outcomes. These measures may complement conventional outcomes such as gait speed in rehabilitation robotics research.

## 2 Materials and methods

### 2.1 Study design

This study was a prospective, randomized controlled pilot study utilizing a single-center, parallel-group design. The trial was conducted at the Department of Physical Medicine and Rehabilitation, Chung-Ang University Hospital in Seoul, Republic of Korea, between March and December 2024. The study was approved in advance by the Institutional Review Board of Chung-Ang University Hospital (IRB No: 2401–020–587). All participants received detailed explanations regarding the study’s purpose and procedures, and provided voluntary written informed consent prior to participation. However, this trial was not prospectively registered in a clinical trial registry prior to participant enrollment, which we acknowledge as a reporting limitation.

### 2.2 Participants

In this study, community-dwelling older adults were recruited. The inclusion criteria were as follows: (1) aged 65–79 years and (2) provided voluntary written informed consent to participate in the study.

The exclusion criteria were as follows: (1) cognitive impairment, defined as a score of less than 24 on the Korean version of the Mini-Mental State Examination (K-MMSE); (2) musculoskeletal disorders that could affect gait function; (3) amputation of any limb; (4) severe internal medical conditions that interfere with activities of daily living; (5) vestibular disorders or episodic vertigo that could increase the risk of falls; and (6) any other conditions deemed inappropriate for study participation by the principal investigator.

Eligible participants were randomly assigned to the robot-assisted training group or the control group in a 1:1 ratio using a computer-generated random number table by a researcher not involved in outcome assessment. Group allocation was concealed until the completion of baseline assessments, after which it was disclosed to the assigned physical therapist, the scheduling researcher, and the participant. Complete blinding of these individuals was not feasible, as participants attended multiple intervention sessions requiring advance preparation of the equipment, and the treating physical therapist was responsible for device fitting and supervision throughout the intervention period. However, to minimize potential bias, the clinical evaluators and the researcher responsible for data analysis remained strictly blinded to group assignments throughout the study period.

### 2.3 Measurement

#### 2.3.1 Assessment schedule and procedures

Assessments were performed at baseline (T1) and post-intervention (T2), with the latter conducted within 1 week after the 3-week intervention. To evaluate the therapeutic carry-over effect of the training rather than the immediate mechanical assistance of the device, all evaluations at T2 for both groups were performed without wearing the robotic suit. To ensure consistency, a single clinical evaluator, who was blinded to the group assignments, conducted all measurements. For safety and fall prevention, a physical or occupational therapist closely accompanied the participants throughout the entire assessment process. Adequate rest periods were provided between tests to minimize the effects of fatigue.

#### 2.3.2 Spatiotemporal gait and plantar pressure analysis

Spatiotemporal gait parameters and dynamic plantar pressure were evaluated using the GAITRite® system (CIR Systems Inc., Franklin, NJ, USA). This portable electronic walkway features an active measurement area 488 cm long and 61 cm wide, embedded with 18,432 pressure sensors arranged in a grid pattern (spaced 1.27 cm apart). The system continuously scans the sensors to capture footfalls dynamically without requiring any attached devices on the participants (Vítečková et al., 2020; Webster et al., 2004).

During the assessment, participants were instructed to walk at their self-selected, comfortable speed. To ensure the measurement of steady-state gait and to eliminate the effects of acceleration and deceleration, participants initiated their walk approximately 1 m before the start of the mat and stopped 1 m past its end, prompted by the verbal command “Get ready, go.”

Each participant performed six consecutive trials under identical conditions. The data extracted from these six trials were averaged to derive a representative value for each participant. Furthermore, the individual values obtained from the left and right feet were averaged to calculate a bilateral mean for all parameters prior to the final analysis. As the study enrolled community-dwelling older adults without hemiparesis or clinically significant neurological deficits, the primary analytical focus was placed on plantar loading distribution and gait patterns, rather than limb-specific asymmetry.

**FIGURE 1:**
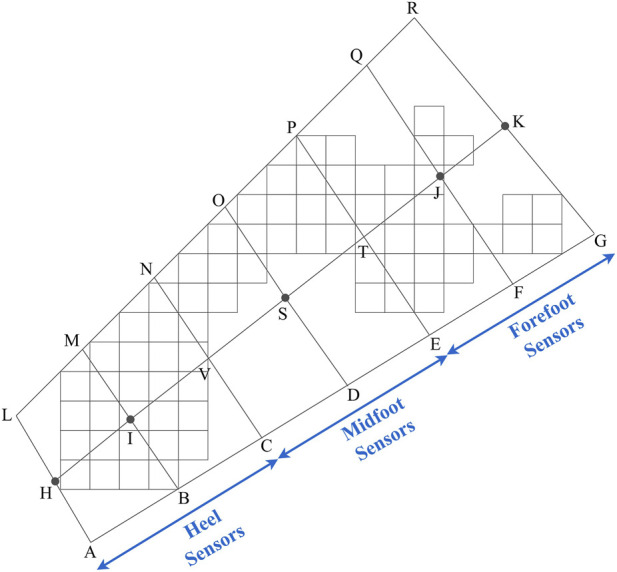
The GAITRite® system divides the footprint into 12 trapezoidal zones, with each group of four trapezoids representing the heel, midfoot, and forefoot zones. The foot shown in the figure is the left foot.

The variables extracted for analysis were broadly categorized into spatiotemporal parameters (e.g., step length, stride length, and phase durations) and plantar pressure characteristics. Self-selected gait velocity (m/s), measured with the GAITRite® electronic walkway, was calculated as the distance traveled divided by the ambulation time across the active sensor area, automatically derived from the GAITRite software. For a detailed evaluation of dynamic plantar loading, the footprint was subdivided into 12 distinct anatomical zones (Figure 1), enabling the specific measurement of contact area and peak pressure distributions within each region (Lim et al., 2016; Guo et al., 2016). Among these, heel and forefoot regions were of particular biomechanical interest given their roles in weight acceptance and propulsion during gait, and changes in contact area and peak plantar pressure across these regions were evaluated as part of the overall plantar loading assessment.

#### 2.3.3 Baseline characteristics and psychological assessments

- Dual-Energy X-ray Absorptiometry (DXA)

To assess the baseline body composition of the participants, DXA (Horizon W; Hologic Inc., Marlborough, MA, USA) was performed. Total body bone mineral density, lean body mass, and fat mass were measured. These data were collected to ensure baseline homogeneity between groups and to objectively confirm that the participants’ physical characteristics were representative of the general older adult population (Kelly et al., 2009; Scafoglieri and Clarys, 2018).

- Beck Depression Inventory (BDI)

The BDI was utilized to evaluate the baseline psychological state and severity of depressive symptoms among the participants. This self-report questionnaire consists of 21 items, each scored on a scale from 0 to 3, with a maximum total score of 63. Higher scores indicate more severe depressive symptoms (Wang and Gorenstein, 2013; Segal et al., 2007).

- Mini-Mental State Examination (MMSE)

The Korean version of the Mini-Mental State Examination (K-MMSE) was administered to screen for cognitive impairment and ensure that participants possessed the cognitive capacity required to safely comprehend and follow the intervention protocols. The assessment evaluates multiple cognitive domains, including orientation, memory, attention, and language, with a maximum possible score of 30 (Kang et al., 1997). As per the study’s inclusion criteria, only individuals scoring 24 or higher were included in the final analysis.

### 2.4 Intervention

#### 2.4.1 Overall protocol

Both groups underwent an identical 3-week intervention program, consisting of two sessions per week (a total of six sessions). To prevent any add-on effects and ensure an equivalent training volume, the protocol was strictly standardized across both groups. Each session lasted 45 min and comprised 20 min of treadmill-based gait training, a 5-min seated rest period, and 20 min of lower-extremity strength training. The only methodological difference between the groups was the application of the robotic device: the robot-assisted training group wore a soft wearable robotic suit during the 20-min treadmill gait training, whereas the control group performed the identical treadmill walking without the device.

#### 2.4.2 Treadmill-based gait training

The 20-min gait training was conducted using a motorized treadmill (AP 2010-2Si; Apsun Inc., Seoul, South Korea). The device was equipped with bilateral handrails and an emergency safety stop function. To ensure maximum participant safety, all individuals wore protective gear, including helmets, knee pads, and elbow pads, throughout the training. The treadmill incline was maintained at 0% (level walking) across all sessions, with exercise intensity modulated solely through speed adjustments. Initially, participants walked at their preferred overground walking speed for adaptation. Subsequently, the treadmill speed was systematically adjusted to maintain an appropriate exercise intensity based on the Borg Rating of Perceived Exertion (RPE) scale. A physical therapist continuously supervised the 20-min sessions to ensure safety and monitor adherence; however, no direct physical assistance was provided to the participants during the training.

#### 2.4.3 Soft wearable robotic suit

**FIGURE 2:**
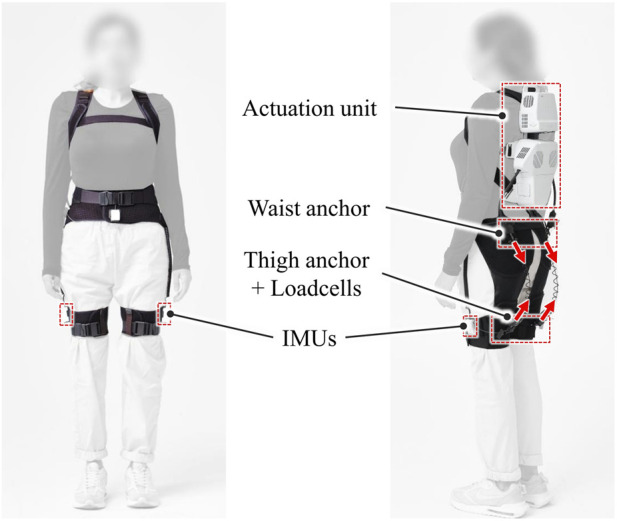
Overview of the H-Medi soft wearable robotic suit.

The device used in this study was H-Medi (HUROTICS Inc., Seoul, Republic of Korea), which provides bilateral hip extension assistance via cable actuation. The device consists of two main components; an actuation unit located at the back, which is worn like a backpack, and a pair of pants that are strapped firmly around the waist and each thigh, as illustrated in Figure 2. When the cable is actuated, tensile force is induced between the waist and thigh anchor points to generate hip extension torque in the sagittal plane. It does not attach rigid links to the lower limbs, to avoid restricting the natural range of motion of the hip joint during usage. The total weight of the device was less than 4.5 kg.

The control algorithm estimates the Gait Cycle Percentage (GCP) in real time using inertial measurement unit (IMU) signals embedded in the device, and provides assistance according to preset timing within each detected gait cycle. In this study, the onset, peak, and offset timings of assistance were set at 4%, 28%, and 42% of the gait cycle based on the timing of maximum hip flexion (Shin et al., 2025; Ding et al., 2018). When expressed in a heel-strike based gait cycle, the onset timing used corresponds to approximately 10% prior to heel contact, and the offset corresponds to approximately 28% of the gait cycle. The assistive force was applied in a half-sine profile.

This assistance timing was designed to support the action of the hip extensors during the around heel contact and during early stance, thereby assisting trunk control and weight acceptance stability. In particular, it was intended to compensate for reduced hip extensor strength commonly observed in older adults and to guide weight transfer more stably during the early stance phase. Outside the assistance window, no active assistance was provided, and the system was maintained in a state in which the Bowden cable became slack, thereby allowing natural lower-limb movement with minimal mechanical resistance.

Previous studies have reported that gait training applying phase-synchronized torque at the hip level can maintain improvements in gait efficiency even after removal of the device, suggesting that wearable gait assistive devices may function not only as walking aids but also as training tools (Martini et al., 2019). Based on these findings, the assistance strategy of this device was not restricted to providing instantaneous assistance during walking, but was also designed to promote the adjustment of gait pattern through repeated training. By providing hip extension assistance around heel contact and weight acceptance, it was intended to allow repeated experience of controlled forward trunk motion and weight transfer, so that a more stable weight-bearing pattern could be reinforced.

Although the device can exert assistance forces of up to 200 N, assistance in this study was applied within a range of 30–80 N, corresponding to approximately 4.5–12 Nm of hip extension torque. Because this research aimed to avoid the robot fully replacing voluntary muscle activation, the magnitude of assistance was set within a range that allowed the user’s active muscle contraction to work in coordination with the assistive force. Therefore, the hip extension assistance strategy applied in this study was considered to have the potential to contribute to changes in weight acceptance characteristics and overall gait pattern through repeated exposure.

#### 2.4.4 Strength training

A 20-min lower-extremity and core strength training protocol was administered identically to both groups. To ensure instructional consistency, the same physical therapist supervised the exercises for each participant throughout the entire 3-week intervention. The training incorporated a comprehensive series of stretching, strengthening, and balance exercises performed across various positions, including supine, side-lying, prone, quadruped, sitting, and standing. The standardized routine included movements such as hamstring and quadriceps stretching, straight leg raises, single-leg bridging, hip abduction and extension, bird-dog exercises, quadriceps setting exercises (QSE), squats, and single-leg stance balance training. Each strengthening exercise was performed for one set of 10 repetitions. The therapist provided individualized manual resistance for specific movements, which was dynamically tailored within the target perceived exertion range.

#### 2.4.5 Exercise intensity and protocol adherence

Throughout the intervention, exercise intensity was strictly maintained at a moderate level, corresponding to a score of 11–13 on the Borg Rating of Perceived Exertion (RPE) scale. To ensure adherence to this target intensity, RPE was systematically assessed four times per session: at the 10-min and 20-min marks during both the strength training and treadmill gait training phases. Based on the participants’ real-time feedback, the therapist adjusted the manual resistance during strength training and modulated the walking speed during treadmill training. For the robot-assisted training group, the assistive force generated by the robotic suit was initiated at 30 N during the adaptation phase. Subsequently, the force was gradually increased in increments of 5–10 N, up to a maximum of approximately 80 N, in strict accordance with the target Borg RPE range.

Strict dropout criteria were established prior to the intervention to maintain protocol integrity. Participants were to be excluded if they missed two consecutive sessions, experienced a significant adverse event, or if the principal investigator deemed their continued participation clinically unfeasible.

### 2.5 Statistical analysis

All statistical analyses were performed using SPSS software (version 30.0, IBM Corp., Armonk, NY, USA). The Shapiro–Wilk test was used to assess the normality of continuous variables. Baseline characteristics between the robot-assisted training group and the control group were compared using the independent t-test or Mann–Whitney U test for continuous variables, and the Chi-square test or Fisher’s exact test, as appropriate, for categorical variables. Within-group differences before and after the intervention were analyzed using the paired t-test or Wilcoxon signed-rank test, according to the distribution of the data.

To compare intervention effects between the two groups, changes in outcome scores (Δ post–pre) were analyzed using the independent t-test or Mann–Whitney U test. Furthermore, a repeated-measures ANCOVA, adjusted for age, body weight and gait velocity as covariates, was conducted. Estimated marginal means were calculated to illustrate divergent pre–post trajectories between the groups. Statistical significance was set at a two-tailed *p*-value <0.05.

## 3 Results

### 3.1 Participants and characteristics

**FIGURE 3:**
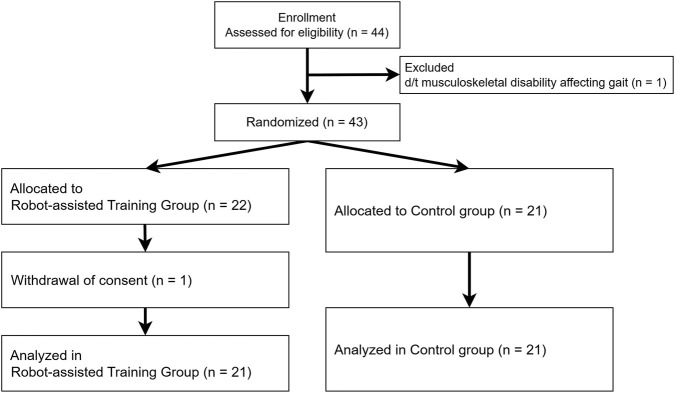
Flow diagram of the study.

As illustrated in Figure 3, a total of 44 participants were initially assessed for eligibility. One participant was excluded prior to randomization due to a musculoskeletal disability affecting gait. The remaining 43 participants were randomly allocated to either the robot-assisted training group (n = 22) or the control group (n = 21). During the intervention period, one participant in the robot-assisted training group was lost to follow-up due to withdrawal of consent. Consequently, a total of 42 participants (robot-assisted training group, n = 21; control group, n = 21) completed the study and were included in the final analysis.

The demographic and clinical baseline characteristics of the participants are presented in Table 1. A total of 42 participants included in the final analysis (robot-assisted training group, n = 21; control group, n = 21) showed no significant differences between groups in age, sex, height, weight, and body mass index. No significant between-group differences were observed in clinical history, including hypertension, diabetes mellitus, hyperlipidemia, osteoporosis, and history of falls. Cognitive and psychological status, assessed by the BDI and MMSE, were similarly distributed between groups. These findings confirm that the randomization process achieved adequate balance in baseline characteristics between the two groups.

**TABLE 1:** Demographic and Clinical characteristics.

Variable	Robot-assisted training group (n = 21)	Control group (n = 21)	*p-*value
Characteristics			
Age (year)	72.19 ± 4.33	72.24 ± 3.79	0.900^a^
Sex (Male/Female)	8/13	6/15	0.513
Height (cm)	156.58 ± 8.89	154.41 ± 7.44	0.397
Weight (kg)	57.93 ± 13.77	56.23 ± 10.28	0.653
BMI (kg/m^2^)	23.39 ± 3.67	23.44 ± 2.95	0.964
Vital signs			
SBP (mmHg)	126.10 ± 17.36	127.19 ± 17.35	0.839
DBP (mmHg)	70.05 ± 9.77	67.62 ± 12.11	0.940^a^
HR (bpm)	74.19 ± 9.81	77.33 ± 8.94	0.284
Clinical history			
Hypertension	9 (42.9%)	7 (33.3%)	0.525
Diabetes mellitus	1 (4.8%)	6 (28.6%)	0.093^b^
Hyperlipidemia	10 (47.6%)	7 (33.3%)	0.346
Osteoporosis	2 (9.5%)	4 (19.0%)	0.663^b^
History of falls	2 (9.5%)	0 (0.0%)	0.488^b^
Cognitive and psychological assessments			
BDI (score)	9.05 ± 6.45	7.43 ± 4.20	0.341
MMSE (score)	28.48 ± 1.29	28.05 ± 1.93	0.680^a^

*p < 0.05.

Values are presented as Mean ± Standard Deviation (SD) for continuous variables or number (percentage) for categorical variables. *BMI,* body mass index; *SBP,* systolic blood pressure; *DBP,* diastolic blood pressure; *HR,* heart rate; *BDI,* beck depression inventory; *MMSE,* Mini-Mental State Examination.

^a^ Calculated using the Mann-Whitney U test (Age, DBP, MMSE).

^b^ Calculated using Fisher’s exact test (Diabetes Mellitus, Osteoporosis, History of Falls).

Unmarked *p*-values were calculated using the independent t-test or Chi-square test.

### 3.2 Between-group differences following the 3-week intervention

#### 3.2.1 Plantar pressure and contact area

Between-group comparisons of change scores (Δ post–pre) revealed significant differences in plantar loading parameters (Table 2).

**TABLE 2:** Between-group differences in plantar pressure parameters (Δ post–pre).

Plantar pressure parameters	Robot-assisted training group (n = 21)			Control group (n = 21)			Mean diff (95% CI)	Effect size	p-value
	Pre	Post	Delta	Pre	Post	Delta			
Heel contact area (AHIB, cm^2^)	8.14 ± 0.74	8.67 ± 1.01	0.52 ± 1.33	8.69 ± 0.89	8.22 ± 0.93	-0.45 ± 1.13	0.98 [0.21, 1.75]	0.79	0.014*
Forefoot contact area (JQRK, cm^2^)	8.05 ± 1.18	7.54 ± 0.84	-0.51 ± 1.12	7.56 ± 0.77	7.86 ± 0.96	0.30 ± 0.98	-0.81[-1.47, -0.16]	-0.77	0.016*
Forefoot peak pressure (ETJF, %)	10.64 ± 1.99	10.17 ± 1.77	-0.49 ± 2.53	10.20 ± 1.86	11.34 ± 2.02	1.13 ± 1.98	-1.62[-3.04, -0.20]	-0.71	0.026*

Values are presented as Mean ± SD.

*p < 0.05.

Independent t-test or Mann-Whitney U test on change score (Δ = post – pre).

Mean diff = mean difference between groups; 95% CI = 95% confidence interval of the mean difference.

Effect sizes were calculated using rank-based effect size or Cohen’s d.

**FIGURE 4:**
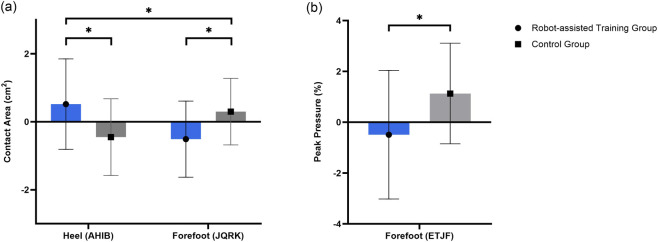
Changes in plantar pressure scores: **(a)** contact area, **(b)** peak pressure in the forefoot. *p < 0.05 indicates a significant between-group difference in change scores (Δ post–pre).

The robot-assisted training group demonstrated a greater increase in heel contact area (AHIB) and greater reductions in forefoot contact area (JQRK) compared with the control group (p = 0.014 and p = 0.016, respectively; Figure 4A).

In addition, forefoot peak plantar pressure (ETJF) decreased more in the robot-assisted training group than in controls (p = 0.026; Figure 4B).

A repeated-measures ANCOVA adjusted for age, body weight, and gait velocity was performed. This analysis revealed significant time × group interactions for plantar loading variables: heel contact area (AHIB; F = 7.273, p = 0.011), forefoot contact area (JQRK; F = 5.042, p = 0.031), and forefoot peak plantar pressure (ETJF; F = 5.535, p = 0.024). No significant main effects of time or covariate interactions were observed.

**FIGURE 5:**
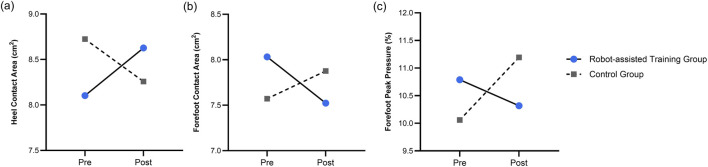
Estimated marginal means of plantar loading outcomes over time by group, adjusted for age, body weight, and gait velocity (Age = 72.21 years, Body weight = 57.08 kg, Gait velocity = 1.11 m/s). **(a)** Heel contact area (AHIB, cm^2^); **(b)** Forefoot contact area (JQRK, cm^2^); **(c)** Forefoot peak pressure (ETJF, %).

As illustrated in Figure 5, estimated marginal means demonstrated divergent pre–post trajectories between the two groups across all plantar loading outcomes, with covariates held at their grand mean values (age = 72.21 years, body weight = 57.08 kg, gait velocity = 1.11 m/s). The robot-assisted training group exhibited an increase in heel contact area and reductions in forefoot contact area and forefoot peak plantar pressure over the intervention period, whereas the control group showed the opposite pattern.

#### 3.2.2 Spatiotemporal gait parameters

With respect to spatiotemporal gait parameters (Table 3), step length (p = 0.005), stride length (p = 0.005), single support (p = 0.032), and swing phase (p = 0.037) were reduced in the robot-assisted training group. An increase in stance phase duration was noted compared with the control group (p = 0.041).

**TABLE 3:** Between-group differences in gait parameters (Δ post–pre).

Gait parameters	Robot-assisted training group (n = 21)			Control group (n = 21)			Mean diff (95% CI)	Effect size	p-value
	Pre	Post	Delta	Pre	Post	Delta			
Step length (cm)	58.85 ± 4.25	57.71 ± 4.32	-1.14 ± 3.54	56.38 ± 5.55	58.34 ± 4.82	1.96 ± 3.25	-3.10 [-5.22, -0.98]	-0.91	0.005*
Stride length (cm)	118.00 ± 8.37	115.71 ± 8.67	-2.29 ± 7.09	113.07 ± 11.09	117.02 ± 9.68	3.95 ± 6.52	-6.24 [-10.49, -1.99]	-0.92	0.005*
Single support (%)	37.80 ± 1.08	37.23 ± 1.50	-0.56 ± 1.29	37.56 ± 1.78	37.90 ± 1.70	0.33 ± 1.29	-0.89 [-1.69, -0.08]	-0.69	0.032*
Swing phase (%)	37.80 ± 1.08	37.25 ± 1.49	-0.55 ± 1.30	37.56 ± 1.79	37.90 ± 1.70	0.33 ± 1.32	-0.87 [-1.69, -0.06]	-0.67	0.037*
Stance phase (%)	62.25 ± 1.09	62.80 ± 1.51	0.55 ± 1.31	62.48 ± 1.78	62.17 ± 1.71	-0.30 ± 1.30	0.85 [0.04, 1.67]	0.65	0.041*

Values are presented as Mean ± SD.

*p < 0.05.

Independent t-test or Mann-Whitney U test on change score (Δ = post – pre).

Mean diff = mean difference between groups; 95% CI = 95% confidence interval of the mean difference.

Effect sizes were calculated using rank-based effect size or Cohen’s d.

## 4 Discussion

This study examined changes in plantar pressure distribution following robot-assisted gait training, compared with conventional gait training, in community-dwelling older adults. After a three-week intervention with a hip extensor–assistive soft wearable robotic suit, the robot-assisted group showed changes in plantar loading, including an increase in heel contact area, as well as changes in spatiotemporal gait parameters compared with the control group. To our knowledge, plantar pressure changes assessed without the device have not been previously reported in community-dwelling older adults.

In the robot-assisted training group, the increase in heel contact area accompanied by reductions in forefoot contact area and peak plantar pressure may suggest a shift towards a heel-centered loading pattern, consistent with more effective use of the heel rocker mechanism. However, because ankle kinematics, tibial progression, and joint kinetics were not directly measured in this study, these plantar pressure findings cannot be interpreted as direct evidence of heel rocker restoration.

During normal gait, energy is efficiently transmitted through a three-rocker mechanism comprising the heel rocker, ankle rocker, and forefoot rocker. Among these, the heel rocker functions as a pivot around the heel during the initial contact and loading response phases, facilitating stable weight acceptance and forward progression of the body (Perry and Burnfield, 2010). In older adults at high risk of falls, weakening of the plantar flexors has been associated with altered weight distribution during the initial contact phase, resulting in excessive concentration of plantar pressure on the forefoot (Pol et al., 2021; Yan et al., 2023), which not only reduces gait efficiency but also exacerbates instability during weight acceptance.

In the present study, the significant increase in the heel contact area during the initial stance phase in the robot-assisted training group may reflect a shift toward a more heel-centered loading pattern and more stable weight acceptance at heel strike. Considering that plantar pressure sensors are sequentially activated from the heel to the toe (Lim et al., 2016), this expanded heel area may suggest the modulated weight-bearing distribution during the initial stance phase (Lee et al., 2017). In contrast, the control group exhibited slight increases in forefoot contact area and peak plantar pressure between the two time points, highlighting a differing short-term trajectory in the absence of robotic assistance.

The observed changes in distal plantar pressure distribution, despite assistance being applied exclusively to the proximal hip joint, may reflect an indirect assistance effect, whereby proximal joint assistance influences the mechanics of distal joints. Both groups performed identical lower-extremity strength training; nevertheless, significant changes were observed exclusively in the robot-assisted group, suggesting that hip extensor assistance may have contributed to the observed gait pattern modifications. The independent contribution of robotic assistance cannot be fully isolated, as joint kinetics and muscle activation were not directly measured. Whether this assistance contributed to attenuating the distal-to-proximal compensatory pattern therefore warrants further investigation with more direct biomechanical assessment. Browne and Franz (2019) reported that ankle power biofeedback can reverse the distal-to-proximal redistribution in older adults, the plantar pressure changes observed here may reflect a comparable proximal-to-distal reorganization.

Lee et al. (2017) reported that even with assistance provided solely at the hip, total foot plantar pressure increased significantly and muscle activation of the rectus femoris and gastrocnemius decreased, suggesting that hip assistance may modify the ankle push-off strategy and thereby contribute to biomechanical efficiency throughout the lower extremity.

In older adults, weakening of the ankle plantarflexors has been associated with a distal-to-proximal redistribution of joint work, in which the hip musculature may be recruited to compensate for reduced distal propulsive capacity, and this compensatory pattern has been suggested as a contributor to increased metabolic cost of gait (Pol et al., 2021; Browne and Franz, 2019; DeVita and Hortobagyi, 2000). Delabastita et al. (2021) further suggested that the hip extensors may be recruited to compensate for diminished ankle propulsion, and that the resulting hip extension moment is an important biomechanical factor related to gait energy expenditure in older adults. Wonsetler et al. (2018) additionally reported that increasing hip extension angle during gait was associated with increases in peak ankle power, ankle plantarflexion work, and anterior propulsive impulse, which may be attributed to the role of adequate hip extension in loading the ankle into dorsiflexion during terminal stance.

Older adults commonly increase the proportion of stance phase and decrease single support time during gait as a compensatory strategy to enhance stability and reduce the risk of falls (Maki, 1997; Inns et al., 2025). Shortening of single support time minimizes the duration spent in a relatively unstable postural state (Hollman et al., 2011). The increase in stance phase proportion in the robot-assisted training group may indicate more stable load transfer during the weight acceptance phase of the study design.

This finding is in line with Park et al. (2013), who reported that reduced single support time and prolonged double support time may serve as a compensatory strategy in older adults with impaired dynamic balance during the loading response and pre-swing phases of gait. The increase in stance phase proportion and reduction in swing phase observed in the robot-assisted training group may similarly reflect a tendency to extend weight acceptance time, potentially contributing to dynamic stability during gait.

Notably, although step length, stride length, single support, and swing phase were reduced in the robot-assisted training group relative to controls, all post-intervention values remained within the normative ranges reported for healthy Korean older adults (Kim et al., 2025). These reductions may therefore reflect a stability-oriented reorganization of the gait cycle within physiologically normal boundaries, rather than functional gait deterioration. Herssens et al. (2020) demonstrated that shorter step length increases the anterior-posterior margin of stability, reducing the risk of backward balance loss, and that decreased single support time limits lateral deviation of the center of mass, thereby preserving medio-lateral stability. These biomechanical principles suggest that the observed spatiotemporal reductions may represent a favorable adaptation for securing dynamic balance in older adults.

Unlike prior studies that measured the immediate assistive effects on plantar pressure while the robot was worn (Lee et al., 2017), the present study assessed plantar pressure without the robot, that may suggest a short-term carry-over effect following repeated training sessions. Whether these changes reflect sustained motor learning or gait pattern reorganization requires confirmation through longer-term follow-up assessment.

The present findings may contribute to a growing body of evidence on the qualitative effects of robot-assisted gait training, encompassing stability and load redistribution. A meta-analysis by Liu et al. (2025) reported that robot-assisted gait training did not demonstrate a significant advantage over conventional training in gait speed in patients with spinal cord injury yet achieved significant improvements in the Timed Up and Go test and qualitative gait outcomes, including reduced trunk sway and improved stable foothold during gait. Rojek et al. (2020) similarly reported that following exoskeleton-assisted gait training in stroke patients, the robot-assisted group demonstrated a plantar load distribution that prevented forefoot overloading, whereas the control group exhibited abnormal forefoot load shifting. A similar pattern was observed in the present study, suggesting repeated hip extensor assistance training may have been associated with more stable weight acceptance from heel strike, reflecting a shift away from a forefoot-dominant plantar pressure pattern.

These changes may also have implications for fall risk. Yan et al. (2023) reported that older adults at high fall risk characteristically exhibit excessive forefoot utilization, whereas low-risk individuals tend to demonstrate more heel-centered loading patterns. The observed shift toward increased heel contact and reduced forefoot loading in the robot-assisted group may be associated with plantar loading patterns that have been linked to lower fall risk in previous research, although direct fall-risk outcomes were not assessed in the present study. These findings extend prior evidence from neurological populations (Lee et al., 2017; Rojek et al., 2020), suggesting that a shift toward a more stable plantar loading pattern may be achievable in community-dwelling older adults after short-term training.

Several limitations of the present study warrant consideration.

First, although this study was approved by the Institutional Review Board prior to commencement, it was not prospectively registered in a clinical trial registry before participant enrollment. As such, the verifiability of prespecified outcomes may be limited.

Second, the soft wearable robotic suit delivered an assistive force (30–80 N, approximately 4.5–12 Nm) intended to cooperate with voluntary muscle activation; however, whether this range was individually optimized was not sufficiently verified. Future studies should establish individualized protocols based on body weight, muscle strength, and gait biomechanics.

Third, as an exploratory pilot study, the sample size was relatively small (n = 42), and generalizability may be limited to individuals with more severely impaired gait, such as frail older adults or those with neurological conditions. Additionally, given that only the robot-assisted group wore the exoskeleton during training, the possibility of performance bias, including novelty or expectation effects associated with wearing the robotic device, cannot be entirely excluded. Future studies with larger and more diverse samples are needed to address these limitations.

## 5 Conclusion

This randomized controlled trial examined the effects of a three-week training program using a cable-driven, hip extensor–assistive soft wearable robotic suit (H-Medi) in community-dwelling older adults. The robot-assisted group showed an increase in heel contact area and reductions in forefoot contact area and peak plantar pressure. Gait cycle phasing also changed, with a prolonged stance phase and a reduced single support time. These changes were observed under device-free assessment.

The findings suggest that hip extensor–assisted training may shift plantar loading toward a more heel-centered pattern. The reductions in selected spatiotemporal parameters likely fall within physiologically normal ranges and may represent adaptive rather than deteriorative changes. Whether these effects reflect sustained gait reorganization requires confirmation in longer-term studies.

Plantar pressure distribution and gait cycle phasing may serve as useful biomechanical outcomes in rehabilitation robotics research, complementing conventional measures such as gait speed.

## Data Availability

The raw data supporting the conclusions of this article will be made available by the authors, without undue reservation.
